# KAS-seq profiling captures transcription dynamics during oocyte maturation

**DOI:** 10.1186/s13048-023-01342-8

**Published:** 2024-01-24

**Authors:** Huiqing An, Xiuwan Wang, Jiashuo Li, Hongzheng Sun, Shuai Zhu, Juan Ge, Longsen Han, Bin Shen, Qiang Wang

**Affiliations:** 1 State Key Laboratory of Reproductive Medicine and Offspring Health, Changzhou Maternity and Child Health Care Hospital, Changzhou Medical Center, Nanjing Medical University, 101 Longmian Rd, Nanjing, 211166 China; 2 Center for Global Health, School of Public Health, Nanjing Medical University, Nanjing, 211166 China

**Keywords:** Oocytes, Meiosis, KAS-seq, ssDNA, Transcription

## Abstract

In fully grown oocytes, the genome is considered to be globally transcriptionally silenced. However, this conclusion is primarily derived from the results obtained through immunofluorescence staining or inferred from the highly condensed state of chromosomes, lacking more direct evidence. Here, by using a kethoxal-assisted single-stranded DNA sequencing (KAS-seq) approach, we investigated the landscape of single-strand DNA (ssDNA) throughout the genome and provided a readout of the activity and dynamics of transcription during oocyte meiotic maturation. In non-surrounded nucleolus (NSN) oocytes, we observed a robust KAS-seq signal, indicating the high transcriptional activity. In surrounded nucleolus (SN) oocytes, the presence of ssDNA still persists although the KAS-seq signal was relatively weak, suggesting the presence of transcription. Accompanying with the meiotic resumption, the transcriptional activity gradually decreased, and global repression was detected in matured oocytes. Moreover, we preformed the integrative genomics analysis to dissect the transcriptional dynamics during mouse oocyte maturation. In sum, the present study delineates the detailed transcriptional activity during mammalian oocyte maturation.

**Supplementary Information:**

The online version contains supplementary material available at 10.1186/s13048-023-01342-8.

## Introduction

During oocyte growth, the chromatin configuration undergoes dynamic modifications, transitioning from a decondensed chromatin configuration typically found in the nucleoplasm of growing oocytes (non-surrounded nucleolus, NSN) to progressively condensed chromatin around the nucleolus (surrounded nucleolus, SN). In mice, growing oocytes are transcriptionally active, accompanied by the accumulation of an enormous quantity of mRNA, but transcription decreases dramatically until the peri-ovulatory stage [1, 2]. Previous investigations into transcriptional activity within mouse oocytes utilizing Br-UTP, Ser2P, and 5-EU incorporation have shown that oocytes with NSN configuration display the elevated levels of transcriptional activity, whereas a shift to the SN configuration is associated with a global transcriptional quiescence [3–5]. With the resumption of meiosis, chromosomes become increasingly condensed, and RNA polymerase disassociate from the chromatin. This leads to a global decrease in Pol II-mediated transcription, which is maintained throughout the subsequent progression of meiosis [4, 6]. Meanwhile, recent studies have revealed some factors that contribute to global transcriptional silencing, such as PCBP1, MLL2 and ZFP36L2 [2, 7, 8]. Nonetheless, the majority of the aforementioned studies deduced oocyte transcriptional repression through immunofluorescence staining or transcriptomics, which are subject to specific limitations. The detection of transcriptional activity via incorporation of 5-EU or Br-UTP presents inherent constraints in sensitivity and poses challenges in quantification. Transcriptomic results are substantially shaped by the intricacies associated with post-transcriptional processing. For instance, a wealth of transcriptomic findings spanning multiple species have revealed a multitude of upregulated transcripts in MII (meiosis II) oocytes relative to GV (germinal vesicle) oocytes [9–13]. Discerning whether the upregulation of these genes stems from transcriptional events or post-transcriptional processing remains a formidable challenge. Thus, the capacity of transcriptomics to faithfully portray the precise transcriptional activity occurring in oocytes is notably constrained. Moreover, ATAC-seq analysis identified tens of thousands of peaks in MII oocytes, suggestive of chromatin accessibility and the potential for concurrent transcription [14]. Hence, the confirmation of transcriptional activity during the process of meiotic maturation necessitates additional validation through alternative technics.

In eukaryotic genomes, DNA is double-stranded, with only one of the strands serving as the transcription template. The initiation of gene transcription starts with the formation of a pre-initiation complex at the promoter region, comprising RNA polymerase and general transcription factors. In active genes, RNA polymerase unwind the double-helical structure of DNA, leading to the formation of single-stranded DNA (ssDNA) regions, often referred to as ssDNA "bubbles" [15]. Hence, ssDNA is not merely important for achieving optimal transcription rates but rather indispensable for the process of gene transcription [16]. In order to comprehensively understand global transcriptional activity, Wu et al. described an approach known as KAS-seq, which enables rapid and highly sensitive labeling of ssDNA through a kethoxal-guanine reaction for subsequent sequencing [17]. In contrast to traditional genome-wide sequencing methods like Pol II Stacc-seq (small-scale Tn5-assisted chromatin cleavage with sequencing) and GRO-seq (Global run-on sequencing) [18, 19], KAS-seq offers the simultaneous assessment of transcriptionally active Pol II, Pol I, and Pol III dynamics, along with non-canonical DNA structures that involve ssDNA in their native context. KAS-seq demonstrates its capability to function efficiently with as few as 1,000 cells due to the rapid, sensitive, and highly specific chemical reactions involving N3-kethoxal and guanine bases within ssDNA. KAS-seq, therefore, enables fast and precise examination of transcription dynamics concurrently, accommodating both low-input and high-throughput applications. Thus, we employed the KAS-seq method to investigate the presence of subtle transcriptional activity during the progression of meiotic maturation.

In the present study, we collected GV (SN and NSN), GVBD (breakdown of germinal vesicle) and MII oocytes, and conducted transcriptional activity assessments using the KAS-seq method. Our primary focus was on characterizing the transcriptional features of mouse oocytes, allowing us to directly elucidate the transcriptional quiescence in meiotic maturation. Additionally, we observed weak gene transcription in SN oocytes, highlighting the significance of these transcribed genes in regulating transcripts stability and mRNA metabolic processes during chromatin configuration transition.

## Materials and methods

### Mouse housing

All animal experiments adhered to the regulations and protocols established by both the local animal ethics committee and the Animal Care and Use Committee of Nanjing Medical University (ethics approval no. 2110009). The research protocol received approval from the Animal Care and Use Committee of Nanjing Medical University. The mice were accommodated in cages maintained under specific pathogen-free conditions and were provided unrestricted access to both water and standard rodent food. Female ICR mice, aged three weeks, were procured from Charles River Laboratories, China Inc.

### Oocyte collection

To acquire Fully Grown Oocytes (FGOs), mice aged 3–4 weeks were subjected to intraperitoneal injections of pregnant mare's serum gonadotropin (PMSG; 7 IU), followed by human chorionic gonadotropin (hCG; 7 IU) administration 46–48 h later. Germinal vesicle (GV) stage oocytes (> 70 μm) were collected 48 h after PMSG treatment. Germinal vesicle breakdown (GVBD) and metaphase II (MII) stage oocytes were collected 3 h and 13 h post hCG injection, respectively. To distinguish between Surrounded Nucleus (SN) and Not Surrounded Nucleus (NSN) oocytes without the need for DNA staining, GV oocytes were cultured in vitro with IBMX (3-isobutyl-1-methylxanthine) for 1 h. Subsequently, GV oocytes were classified into two groups based on the presence or absence of a visible Perivitelline Space (PVS). Oocytes with PVS were designated as SN oocytes, while those lacking PVS were categorized as NSN oocytes. For MII oocytes, the zona pellucida and first polar body was carefully removed through treatment with Pronase E (Solarbio P8360).

### Low-input KAS-seq

Oocytes were labeled with N3-Kethxal, and labeled genomic DNA was subsequently extracted using the Quick-DNA Microprep Plus Kit (Zymo D4074).After labeling, gDNA was fragmented using Tn5 transposase (Vazyme TD501) under incubation conditions of 37 °C and 500 rpm for 30 min. Following fragmentation, biotinylation labeling was performed using DBCO-PEG4-biotin (DMSO solution, Sigma 760,749). The biotinylated DNA was then cleaned up using DNA Clean & Concentrator-5 kit (Zymo, D4013), and 5 µL of labeled DNA was retained as input, while the remaining 50 µL of DNA was used for enrichment. For bead-based enrichment, 1 × Binding and Wash buffer was used to wash 5 µL of Dynabeads™ MyOne™ Streptavidin C1 (Thermo 65,001) three times. Pre-washed beads were then resuspended in 2 × Binding and Wash buffer. The beads were mixed with the 50 µL fragmented DNA obtained from the previous steps and rotated slowly at room temperature for 15 min. After incubation, the mixture was placed on a magnetic rack to remove the supernatant, and the beads were washed five times with 1 × Binding and Wash buffer. DNA-conjugated beads and their corresponding inputs were used for library PCR using i5 and i7 index primers (Vazyme TD205) and VAHTS HiFi Amplification Mix (Vazyme N616). The PCR reactions were initiated with a 5 min incubation at 72 °C, followed by 10 min at 95 °C, and then amplified for 14 cycles (10 s at 98 °C, 30 s at 60 °C, 1 min at 72 °C). Finally, the libraries were cleaned up by using DNA Clean & Concentrator-5 kit (Zymo, D4013).

### KAS-seq data processing

Paired-end reads of 150-bp were generated on the Illumina novaseq 6000 platform in this study (sequenced by Annoroad). For KAS-seq data, raw reads were trimmed using trim galore (v0.6.10) (parameters: –quality 20 –phred33 –length 30 –stringency 3 –paired) and mapped to mouse reference genome (GRcm38) using Bowtie2 (v2.5.1) (parameters: -X 2000 -I 10). All unmapped reads, non-uniquely mapped reads and PCR duplicates were removed. The signal tracks and heatmap were both generated by deeptools (v3.5.2). The MACS2 (macs2 2.2.7.1) were used for peak calling (parameters: –broad). HOMER (v4.10) was used for motif enrichment analysis. Annotation of peaks was performed using Staccseeker (v1.36.0).

### Quantitative real-time PCR

The mRNA levels of *Padi6, Gdf9, Bmp15,* and *Zp2* were assessed through quantitative real-time polymerase chain reaction (qRT-PCR) employing the comparative CT method, as previously outlined in our research. Total cellular RNA was extracted from a pool of 50 oocytes from the specified experimental group, employing the RNAprep Pure Micro Kit (TIANGEN DP420). Subsequently, first-strand cDNA was synthesized following the manufacturer's protocols using the HiScript III RT SuperMix for qPCR (Vazyme R323).Real-time PCR assays were conducted with SYBR Green in a final reaction volume of 10 μL, utilizing an ABI QuantStudio™7 Flex PCR system (Applied Biosystems). GAPDH served as the internal reference control for all samples, and each experiment was carried out in triplicate. The primer sequences are provided bellow:

- *Padi6*-F: TGGTAGGCATGGAAATCACCT;
- *Padi6*-R: GACGGAGCTAGAGATGTGGAT;
- *Gdf9*-F: TCTTAGTAGCCTTAGCTCTCAGG;
- *Gdf9*-R: TGTCAGTCCCATCTACAGGCA;
- *Bmp15*-F: TCCTTGCTGACGACCCTACAT;
- *Bmp15*-R: TACCTCAGGGGATAGCCTTGG;
- *Zp2*-F: GTGGCAGAGGAAAGCATCTGT;
- *Zp2*-R: GACTGAGGAAGGCTTACTGAGT.

### EU staining

The EU stock solution was diluted to 100 mM with ddH2O. Subsequently, the EU solution (100 mM) was diluted 1:100 with M2, and SN/NSN oocytes were added, followed by incubation at 37 °C for 45 min. The oocytes were then placed in 4% PFA at 4 °C overnight. Subsequently, the oocytes were permeabilized by incubation in 0.5% Triton X-100 for 15 min. Afterward, they were washed three times with PBST, with each wash lasting 3–5 min. Next, the oocytes were incubated for 30–60 min at room temperature with the Click-iT reaction cocktail (Click-iT™ RNA Alexa Fluor™ 488 Imaging Assay Kit, ThermoFisher C10329). Following this, they were washed once with a raise buffer for 3–5 min and then washed once with a wash buffer for 3–5 min. DNA staining was performed using Hoechst 33,342 at a dilution of 1:1000 in PBST for 15 min. Finally, the oocytes were washed three times with a wash buffer, with each wash lasting 3–5 min.

### RNA-seq

Oocytes were added into 1 μL of Sample Buffer (prepared by adding 2 μL of RNase Inhibitor (Vazyme R301-01) to 18 μL of Lysis Buffer containing 0.2% (vol/vol) Triton X-100). Care was taken to ensure that the liquid volume in the micropipette was approximately 0.5–1 μL, and then it was supplemented with Nuclease-free H2O to a total volume of 3.5 μL. Subsequently, the cDNA synthesis reaction was prepared as follows: the above sample (3.5 μL) was mixed with 1 μL of Oligo(dT) VN Primer (1 μM) and 1 μL of dNTP Mix (Thermo R0912). The reaction mixture was incubated at 72 °C for 3 min and immediately placed on ice. Next, the reverse transcription reaction was prepared as follows: the above product (5.5 μL) was mixed with 2 μL of First Strand Buffer (Vazyme R721-01), 0.5 μL of DTT (Vazyme R721-01), 0.5 μL of RNase Inhibitor, and 1 μL of Sc Reverse Transcriptase (Vazyme R721-01). The reaction was carried out in a PCR machine with the following program: 42 °C for 90 min; 50 °C for 2 min, 42 °C for 2 min (return to the second step, repeat for 2–11 cycles); 70 °C for 15 min; and finally, hold at 4 °C.Subsequently, the cDNA amplification reaction was prepared as follows: 10 μL of the first-strand cDNA synthesis product was mixed with 2 μL of Nuclease-free H2O, 0.5 μL of ISPCR Primer (5 μM), and 12.5 μL of 2 × Amplification Mix (Vazyme N616-01). The reaction was carried out in a PCR machine with the following program: 98 °C for 1 min; 98 °C for 10 s, 65 °C for 15 s, 72 °C for 6 min (return to the second step, repeat for 15 cycles); 72 °C for 5 min; and finally, hold at 4 °C.The cDNA amplification products were purified using VAHTS DNA Clean Beads (Vazyme N411). Subsequently, quality control of the cDNA amplification products was performed, including measuring cDNA concentration using Qubit 4 and assessing cDNA fragment distribution using Qsep 400.Following this, sequencing libraries were prepared using the TruePrep DNA Library Prep Kit V2 for Illumina (Vazyme TD503). The sequencing libraries were then purified using VAHTS DNA Clean Beads (Vazyme N411). And then, quality control of the sequencing libraries was performed, including measuring library concentration using Qubit 4 and assessing library fragment distribution using Qsep 400.

### RNA-seq data processing

For RNA-seq data, adaptors and low-quality bases were removed with trim_galore (v0.6.10) and sequences were mapped to mouse reference genome (GRCm38) using HISAT2 (v2.2.0) with gencode annotation (vM25). Read quantification was carried out with FeatureCounts (v2.0.6) (parameters: -t exon -g gene_id -Q 10 –primary -s 0 -p). Differentially expressed genes (DEGs) were determined using DESeq2 (v1.40.2) (parameters: > twofold change with adjusted *P* < 0.01).

### ATAC-seq and Pol II Stacc-seq data processing

For Pol2 and ATAC-seq data, raw reads were trimmed using trim_galore and mapped to mouse reference genome (GRcm38) using Bowtie2 (v2.5.1) [20] (parameters: -X 2000 -I 10). All unmapped reads, non-uniquely mapped reads and PCR duplicates were removed. The signal tracks and heatmap were both generated by deeptools (v3.5.2) [21]. The peaks were identified by using MACS2 [22].

### Statistical analysis

Statistical data were presented as the mean ± SD in bar charts. For boxplots, middle lines indicated the median, the boxes indicated the 25th/75th percentiles. KAS-seq and RNA-seq was repeated twice. qPCR and immunofluorescence experiments were repeated at least three times, and* P* values were determined by two-sided Student’s t-test using GraphPad Prism (version 8.0.1). Significant differences were shown with “***” for indicating *P* < 0.001. “#” denotes no significance.

## Results

### Genome-wide profiling of ssDNA during meiotic maturation

**Fig. 1:**
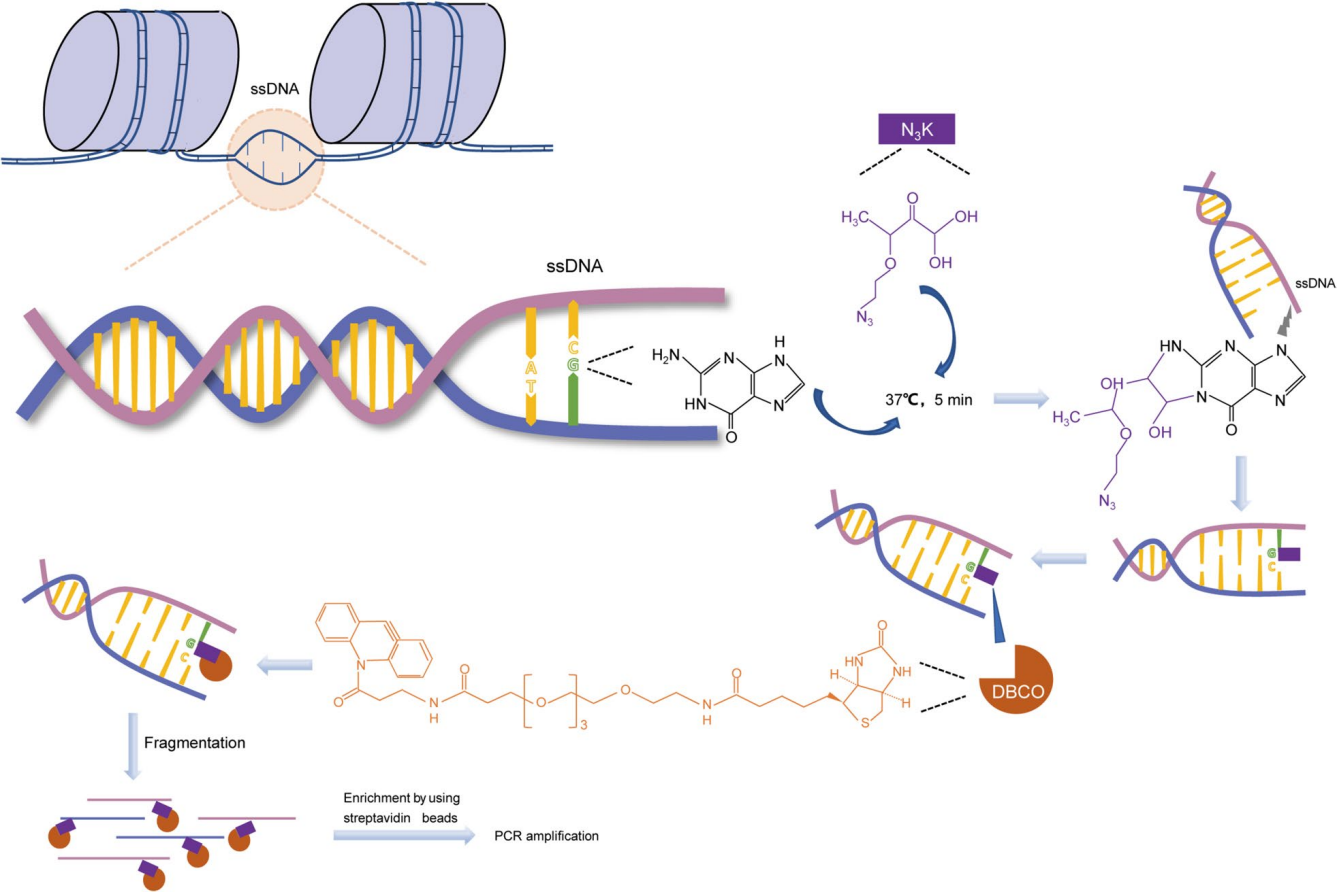
KAS-seq probes regions of single-stranded DNA. N3-kethoxal (indicated by the purple box) selectively engages with single-stranded guanine residues within the genomic DNA (resolved by DNA-binding proteins), subsequently amenable to biotinylation (highlighted in orange) and subsequent enrichment for sequencing

**Fig. 2:**
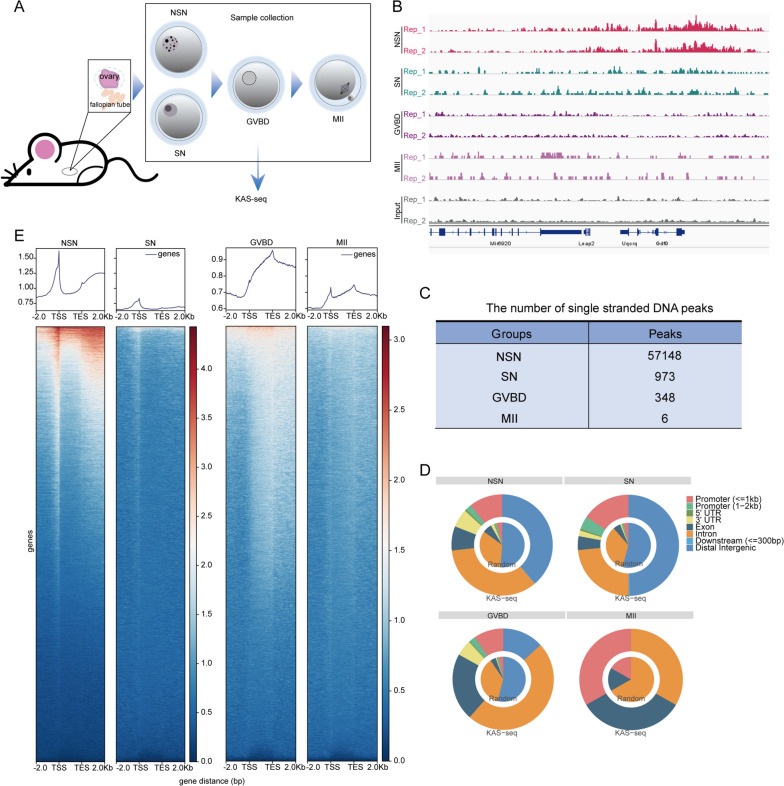
KAS-seq profile of mouse oocyte maturation. **A**. Schematic overview of the workflow for KAS-seq profiling of mouse oocytes isolated at GV (SN and NSN), GVBD, and MII stages. **B**. A snapshot from UCSC browser views showing KAS-seq peaks in GV (SN and NSN), GVBD and MII oocytes. **C**. Number of ssDNA peaks detected by KAS-seq in GV (SN and NSN), GVBD and MII oocytes. **D**. The distribution of KAS-seq peaks across the genome was analyzed. KAS-seq signifies the percentage of overlap between KAS-seq peaks and various genomic features, while Random represents the percentage of overlap between randomly generated regions, matching the number and length of actual peaks, with various genomic features. **E**. KAS-seq signal distribution at gene-coding regions in GV (SN and NSN), GVBD and MII oocytes

Azide-tagged kethoxal (N3-kethoxal), a modified kethoxal [23], retains not only its elevated reactivity and specificity towards guanine residues within single-stranded nucleic acids, but also provides a bio-orthogonal handle that can be easily functionalized with biotin (Fig. 1). Using the optimized KAS-seq assay, we systematically performed the library construction and ssDNA analysis of GV, GVBD, and MII oocytes (Fig. 2A, Supplementary Table 1). The experiment was conducted in two replicates with 300 oocytes per sample, a remarkable reduction in sample quantity compared to previous study [17]. KAS-seq exhibits robustness and reproducibility, demonstrating a notable enrichment efficiency and strong correlation, as well as a substantial overlap of peaks across replicates (Figs. S1, S2 and Fig. 2B). As expected, the ssDNA signal is enriched in transcriptionally active NSN oocytes, with over 57,000 peaks detected. With the transition in chromatin configuration, there is a sharp decline in ssDNA signals in SN-configured oocytes. Simultaneously, the ssDNA signal further attenuates accompanying with meiotic resumption (Fig. 2C). We particularly observed that in MII oocytes, only six peaks were detected (Fig. S3A), which represent extremely small ssDNA regions relative to the entire mouse genome. In NSN oocytes, KAS-seq reads are considerably enriched at gene-coding regions, especially at promoter regions and transcriptional termination areas. However, the six peaks in MII oocytes are primarily located within promoters, introns, and exons, with no distribution in transcription termination regions (Fig. 2D). These results suggest that the transcriptional activity in MII oocytes is exceedingly low. In NSN oocytes, KAS-seq profiles of gene-coding regions unveiled distinct features, including a prominent and sharp peak proximal to the transcription start site (TSS), comparatively faint and extensive signals spanning the entire gene body, and a robust yet broad peak emerging from the transcription termination site (TTS) extending into downstream regions (Fig. 2E). The distribution pattern of KAS-seq signals in NSN oocytes is consistent with that observed in somatic cells [17], serving as a direct indicator of increased transcriptional activity. In SN oocytes, although the KAS-seq signal is weak, the distribution pattern of ssDNA regions within gene-coding regions was similar to NSN oocytes(Fig. 2C, D). Indicating the presence of relatively weak gene transcription in SN oocytes. Simultaneously, we observed that in GVBD oocytes, KAS-seq profile on gene-coding regions is distinct from that in other stages. The ssDNA signal is relatively weak around the TSS region, but it gradually intensifies along the gene body, reaching a strong and broad peak near the TTS (Fig. 2E, Fig. S3B and C). This observation suggests that following meiotic resumption, transcription gradually ceases until reaching the GVBD stage. As transcription progresses, Pol II migrates from the TSS region towards the TTS region. Meanwhile, due to chromatin condensation, there is a reduction in de novo transcription, which leads to the gradual accumulation of ssDNA signals in the TTS region.

### Integrative genomics analysis of transcriptional activity in SN and NSN oocytes

**Fig. 3:**
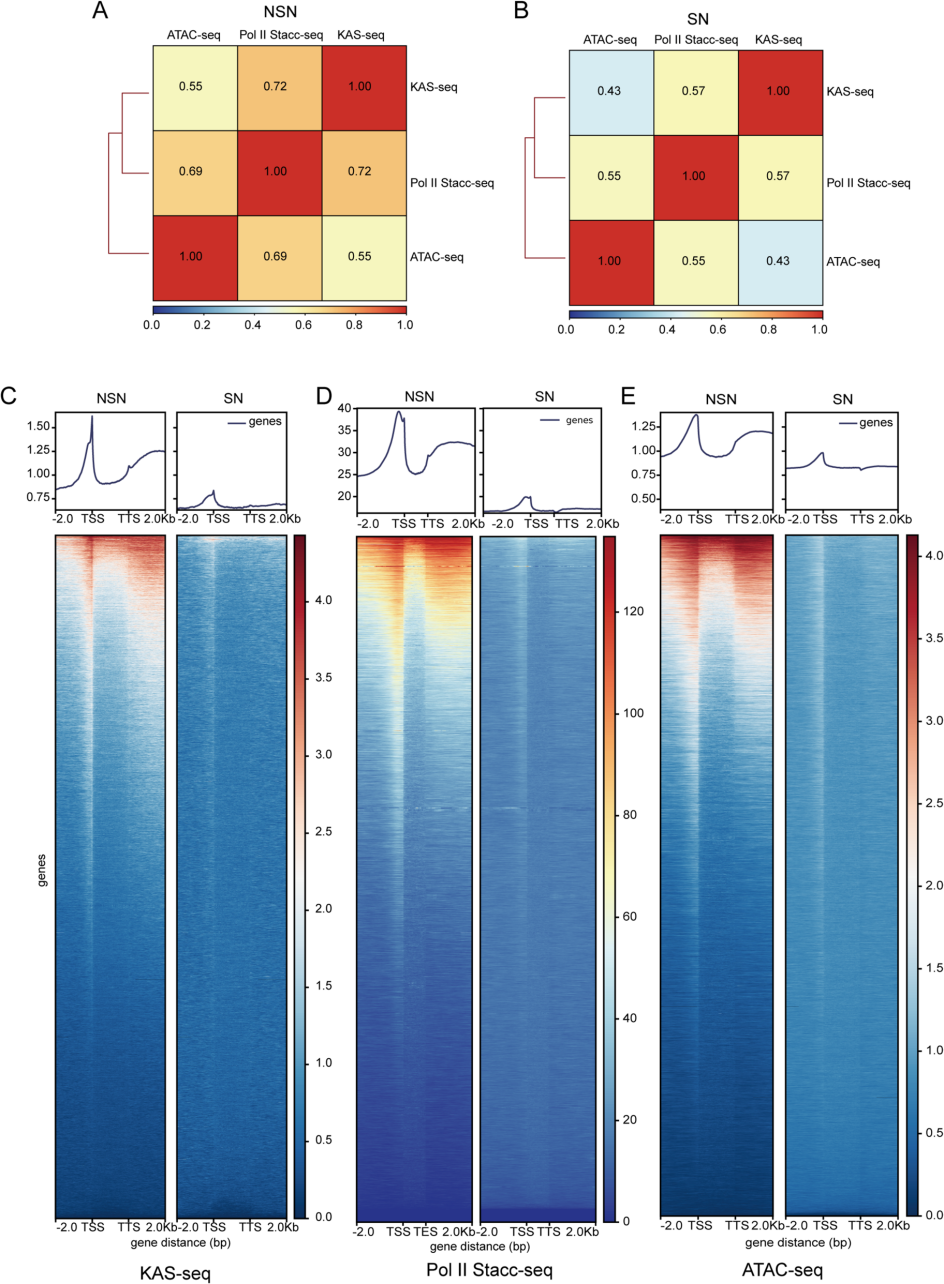
Integrative genomics analysis of transcriptional features in SN and NSN Oocytes. **A**, **B**. Genome-wide Pearson correlation heatmap depicting the association between KAS-seq, ATAC-seq and Pol II Stacc-seq. **C**-**E**. Heatmap showing KAS-seq, Pol II Stacc-seq and ATAC-seq signal distribution at gene-coding regions, respectively. Regions 2 kb upstream of TSS and 2 kb downstream of TTS were shown

Previous studies showed that SN oocytes possess a higher developmental potential, as they are better equipped to support early embryonic development than NSN oocytes [24, 25]. Understanding the intricate transcriptional dynamics between NSN and SN oocyte is fundamental to elucidate the molecular processes underlying their developmental potentials. We previously performed the analysis of chromatin accessibility by ATAC-seq in SN and NSN oocytes (data not published). Pol II Stacc-seq was also employed to investigate the differences in transcriptional activity between SN and NSN oocytes [26]. Here, we conducted a comparative analysis between KAS-seq and these two sequencing techniques to delve deeper into the transcriptional characteristics SN nd NSN oocytes. This allowed us to elucidate whether KAS-seq technology offers advantages over the other two methods. Initially, we collected NSN and SN oocytes using the previously established method (Fig. S4A). The results indicate that this method effectively distinguishes NSN from SN oocytes (Fig. S4B). We subjected these oocytes to KAS-seq analysis. Subsequently, we performed a comparative analysis by juxtaposing the results obtained through KAS-seq with those from ATAC-seq and Pol II Stacc-seq (Fig. 3A, B). In both NSN and SN oocytes, KAS-seq results correlate well with the findings from Pol II Stacc-seq. This observation suggests that KAS-seq, in comparison to ATAC-seq, provides a more accurate representation of the transcriptional pattern in oocytes. Through an analysis of signals within gene-coding regions, we observed that in NSN oocytes, both Pol II signals and ATAC signals are considerably enriched at gene-coding regions, especially at gene promoters and transcription termination regions, exhibiting a distribution pattern consistent with the features observed in KAS-seq signals (Fig. 3C**-**E). In SN oocytes, the signals in the gene coding regions for KAS-seq, Pol II Stacc-seq, and ATAC-seq have all experienced a drastic reduction compared to NSN oocytes. However, there are differences in the signals identified by these three techniques in SN oocytes, particularly in the case of KAS-seq and ATAC-seq. As shown in the peak plots, the signal intensity is primarily concentrated around the transcription start site TSS, with a relatively weak signal increase near the TTS, exhibiting a pattern similar to that of Pol II Stacc-seq signals. In contrast to KAS-seq and Pol II Stacc-seq, the ATAC-seq signal is predominantly localized around the TSS with a robust signal but not around TTS region in SN oocytes. Additionally, quantitative statistical analysis of ATAC-seq peaks signals showed that 4,968 genes in the accessible chromatin region were covered in SN oocytes. While, the peaks density within the gene coding regions of SN oocytes is relatively weak, particularly in the TTS region, which is consistent with the condensed chromatin state of SN oocytes. This minimal chromatin accessibility makes it challenging to determine the presence of transcriptional activity in SN oocytes. KAS-seq data provide a more objective reflection of the transcriptional characteristics of SN oocytes. We can observe that while the transcriptional activity in SN oocytes is reduced compared to NSN oocytes, with a limited number of genes showing transcription, in line with the results of 5-ethynyl uridine staining (EU staining, a method to visualize newly synthesized RNA) (Fig. S4C, D). Subsequently, we conducted a functional enrichment analysis of the upregulated transcripts identified by RNA-seq in SN oocytes. We found that the upregulated genes in SN oocytes were enriched in processes related to meiotic nuclear division, chromosome organization, and sister chromatid cohesion (Fig. S4E), all of which are crucial for the resumption and maturation of meiosis. In summary, KAS-seq method, compared to ATAC-seq technique, offers a superior reflection of the transcriptional activity in oocytes.

### KAS-seq data and transcriptome results exhibit differences

**Fig. 4:**
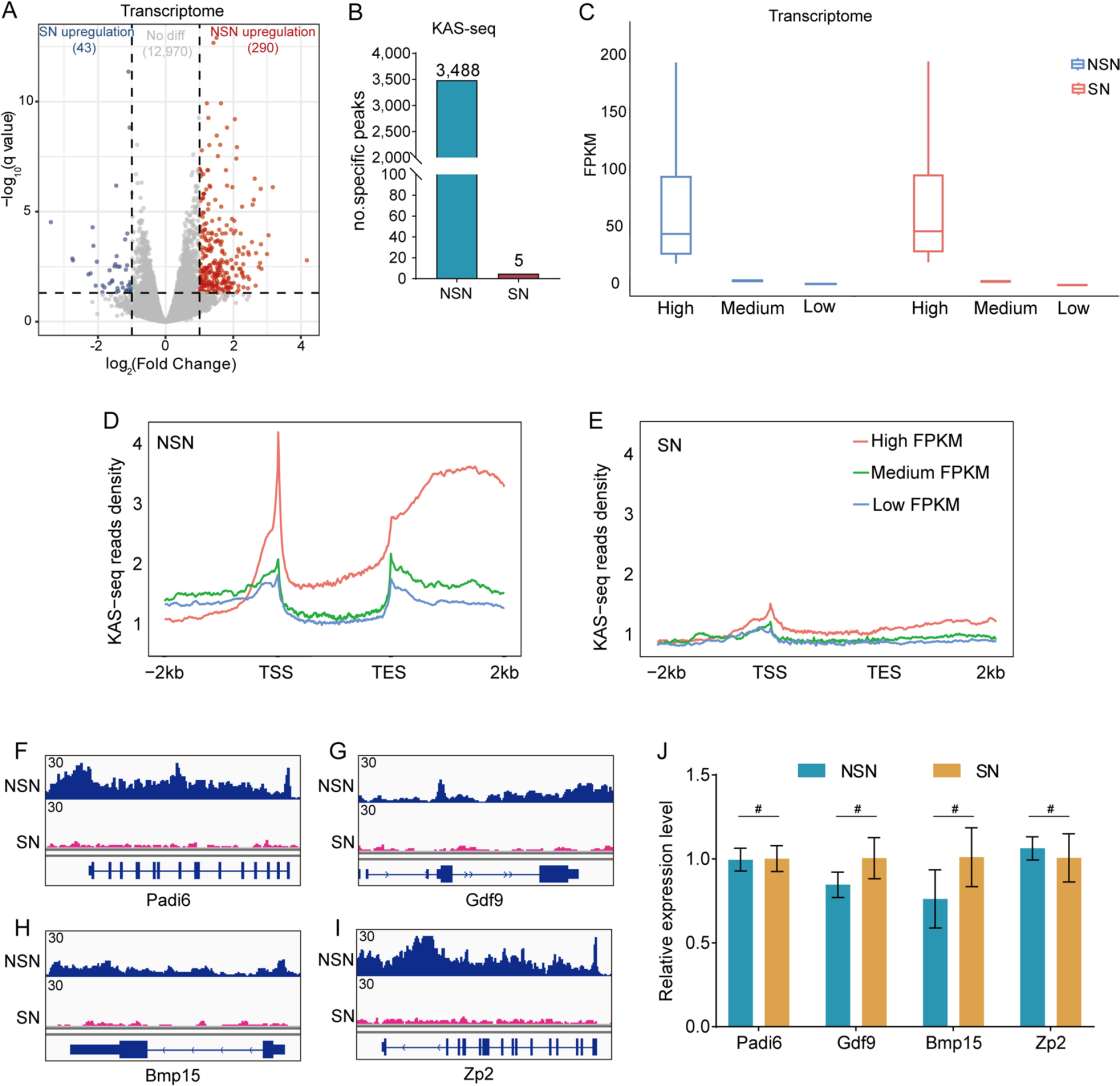
Integration analysis of KAS-seq and transcriptome in SN and NSN oocytes. **A**. Volcano plots comparing differentially expressed genes between SN and NSN oocytes (*p*.adjust < 0.05, Fold chang > 2). **B**. Bar charts showing the unique peaks identified in SN and NSN oocytes. **C**. Genes were grouped according to different expression levels based on transcriptome. **D**, **E**. KAS-seq reads density at gene-coding regions of genes with different expression levels (defined by transcriptome) in SN and NSN oocytes. **F**-**I**. Genome browser snapshot of KAS-seq peaks in SN and NSN oocytes near *Padi6, Gdf9, Bmp15 and Zp2*, respectively. **J**. Bar charts showing the relative expression levels of *Padi6, Gdf9, Bmp15 and Zp2* quantified by RT-qPCR in SN and NSN oocytes. # *p* > 0.05

In the above results, we investigated the differences of transcriptional activity between SN and NSN oocytes through KAS-seq profile, we next explored the correlation between KAS-seq and the transcriptome. Firstly, transcriptome analysis was performed upon chromatin configuration transition. Despite the differences in transcriptional activity between SN and NSN oocytes, their transcriptome differences are minimal, and the RNA-seq results show highly positive correlation with each other (Fig. S5A). There are only 333 differential transcripts (FC > 2, adjust *p* < 0.01) between SN and NSN oocytes out of tens of thousands of transcripts (Fig. 4A), which is inconsistent with the differences in their transcriptional activity. However, there is a substantial difference in the specific peaks identified in SN and NSN oocytes through KAS-seq profile (Fig. 4B, S5B). We then ranked all transcripts of SN and NSN oocytes into three groups according to their expression levels based on RNA-seq data [17] (Fig. 4C), and showed that the strength of KAS-seq signals increase notably in genes with high expression levels in NSN oocytes (Fig. 4D). This suggests that in NSN oocytes, the KAS-seq results are consistent with the RNA-seq data and can directly measures transcripts generation. In contrast to NSN oocytes, where highly abundant transcripts exhibit a strong KAS-seq signal, SN oocytes show a notably weak KAS-seq signal corresponding to highly abundant transcripts. Therefore, during the chromatin configuration transition from NSN to SN, there is an inconsistent dynamic change between the transcript’s abundance and the intensity of KAS-seq signals (Fig. 4E). We subsequently validated the correlation between KAS-seq data and transcripts. The quantitative PCR results indicate that gene expression is not significantly different between SN and NSN oocytes, although there is a substantial difference in KAS-seq signals (Fig. 4F, J). The above results indicate that during chromatin configuration transition, although there are significant changes in the dynamic of single-stranded DNA, accompanied by a decrease in transcriptional activity, the transcripts stored in oocyte cytoplasm remain relatively stable.

### ssDNA dynamics upon chromatin configuration transition

**Fig. 5:**
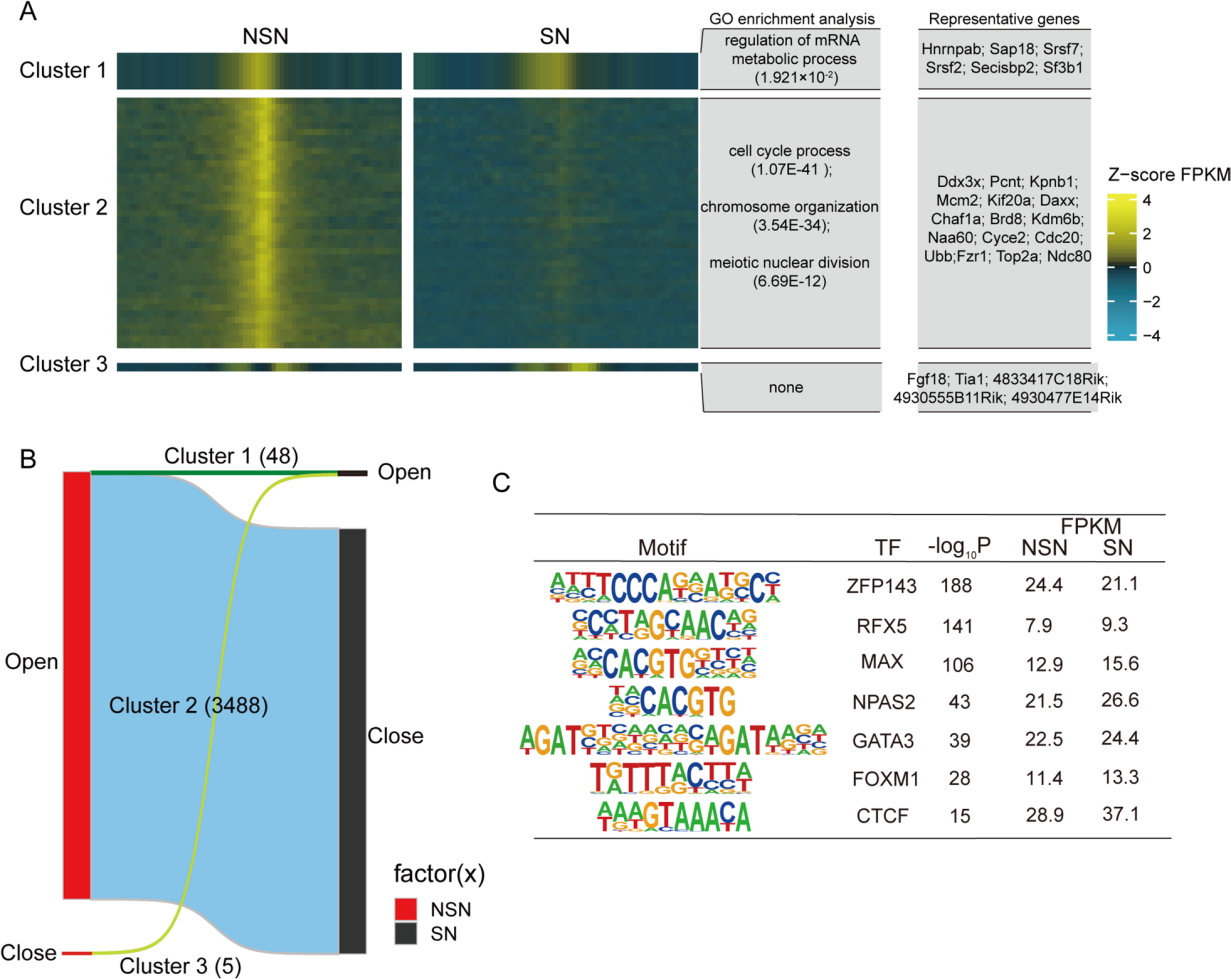
The dynamics of KAS-seq signals in SN and NSN oocytes. **A**. Heatmaps showing the dynamics of KAS-seq signals in SN and NSN oocytes. The three clusters are displayed in the left panel, and the representative genes and biological processes of each cluster are shown in right panel. **B**. Alluvial plots showing the global dynamics and the number of KAS-seq signals of cluster 1(open to open), cluster 2 (open to close) and cluster 3 (close to open) in NSN and SN oocytes. The TSS ± 2 Kb genomic regions were clustered by using deepTools (plotHeatmap –kmeans 4). **C**. Transcription factor motifs identified from promoter KAS-seq signals in both SN and NSN oocytes

To further elucidate the potential function of KAS-seq signals that were gained, lost, or maintained during the transition in chromatin configuration, ssDNA signals in SN and NSN oocytes were categorized into four distinct clusters by using *k*-means algorithms (Supplementary Table 2): two stage-open domains (cluster 1), NSN-open domains (cluster 2), SN-open domains (cluster 3) and two stage-close domains (cluster 4) (Fig. S5C). Given that cluster 3 comprises only five domains, we conducted a more focused analysis on the domains within cluster 1 and cluster 2 (Fig. 5A, B). Gene Ontology (GO) analysis indicated that domains in cluster 1 was deposited for genes involved in regulation of mRNA metabolic process; whereas domains in cluster 2 were deposited for genes in cell cycle process, chromosome organization and meiotic nuclear division (Fig. 5A). These results indicate that genes with sustained expression in both SN and NSN oocytes may be involved in maintaining transcript stability. Due to the shared domains in both SN and NSN oocytes, we asked whether the common domains harbor motifs for transcription factors that involved in the sustained transcription of these genes. Notably, the ZFP143 factor is strongly enriched in the shared domains, and it is also highly expressed in both SN and NSN oocytes (Fig. 5C). Previous research has shown that the ZFP143 knockout leads to infertility, indicating a critical role for ZFP143 in the reproductive process of mice. However, the specific mechanism still requires further exploration.

## Discussion

The generation of ssDNA is a prerequisite for transcription initiation. For the first time, we have dissected the transcriptional features during oocyte maturation from this perspective. Throughout the process of oocyte growth, the nucleus undergoes dynamic morphological changes, accompanied by a pronounced reduction in transcriptional activity as the oocytes approach full size. Simultaneously, it is widely acknowledged that a state of global transcriptional quiescence ensues immediately prior to the resumption of meiosis. In this study, we employed KAS-seq technology to unveil the presence of transcriptional activity in highly condensed chromatin within SN oocytes, contrary to prior research findings [4]. Simultaneously, during the process of meiotic maturation, the gradual reduction in ssDNA peaks is substantiated by the predominant concentration of ssDNA signals in the TTS region of GVBD oocytes, with relatively weaker signals observed in the TSS region. Furthermore, our investigations into MII oocytes revealed the genome to be in a state of complete transcriptional quiescence.

To gain insights into global transcriptional regulation, various genome-wide sequencing approaches have been developed. These approaches aim to investigate RNA polymerase occupancy (e.g., Stacc-seq) or to assess the presence and abundance of nascent RNA molecules [18, 27]. The analysis of nascent RNA generally relies on techniques such as run-on assays, metabolic labeling [28, 29], and RNA enrichment associated with Pol II or chromatin [26]. While these methods offer valuable insights, they come with certain limitations. Run-on-based techniques and Pol II-associated RNA enrichment typically necessitate a substantial number of cells as the starting material. Additionally, Pol II Stacc-seq may not provide clear discrimination between RNA polymerases that are merely bound and those that are actively engaged in transcription. Furthermore, metabolic labeling may not accurately quantify transient and low-abundance RNA species. The KAS-seq technique is developed based on the accessibility of ssDNA. It exhibits high sensitivity in detecting ssDNA signals, requiring only a thousand cells for analysis. Moreover, it offers the advantage of a straightforward workflow, with sample library construction being completed in just a few hours. Oocytes in female mammals are extremely precious as they cannot be readily obtained in large quantities. Therefore, the use of the highly sensitive KAS-seq profile to detect the transcriptional activity is a suitable choice. In this study, we were able to obtain high-quality ssDNA database using as few as 300 oocytes, and the entire library construction process took approximately 8 h. This efficiency proves to be highly advantageous for our analysis of the true transcriptional landscape during meiotic maturation.

MII oocytes are typically regarded as being in a transcriptionally silent state due to their highly condensed chromosomes, but there has been a persistent lack of direct evidence to conclusively demonstrate this. Previous RNA-seq studies focused on MII oocytes have indicated a substantial upregulation of transcript expression compared to GV oocytes [9]. These findings introduce a contradiction, suggesting that we cannot conclusively infer a state of transcriptional silence in MII oocytes solely from transcriptomic results due to post-transcriptional processing of transcripts and certain limitations in library preparation techniques. KAS-seq technology, on the other hand, can directly detect ssDNA in the genome, which serves as a prerequisite for transcriptional activity. Our results demonstrate that only 6 ssDNA peaks were detected in the genome of MII oocytes, and these peaks exhibited atypical distribution characteristics. The presence of such a limited number of ssDNA peaks can provide evidence to support the notion that the genome of MII oocytes is in a state of transcriptional silence. While transcription is largely silenced in MII oocytes, post-transcriptional regulation can still influence the abundance and stability of mRNA molecules. Specific dormant RNAs, such as *Mos*, *Plat*, and *Cnot7*, exhibit either short or absent poly(A) tails and maintain stability throughout oocyte growth [30]. Upon the resumption of meiosis, these RNAs undergo rapid polyadenylation [31, 32]. Therefore, the length of mRNA poly(A) tails may be an important factor influencing the outcomes of transcriptome analysis.

In mammals, extensive chromatin reorganization is essential for oogenesis and subsequent meiotic maturation. In oocytes, the NSN chromatin configuration is associated with elevated transcriptional activity, whereas a concurrent transition to the SN configuration corresponds to a global repression of transcription. In this study, we employed KAS-seq profile to detect the presence of several hundred ssDNA peaks in SN oocytes. Furthermore, it was observed that the genes deposited with these peaks play pivotal roles in the regulation of meiotic cell cycle, meiotic nuclear division, and sister chromatid cohesion processes. These findings suggest that the genes exhibiting continuous expression in SN oocytes play a pivotal role in the meiotic maturation. While the transcriptional activity dramatically decreases, transcripts, however, exhibit relatively small differences during the transition from NSN to SN configurations. To uncover potential mechanisms, we conducted gene ontology analysis of the genes with sustained transcription in both NSN and SN oocytes. We found that these genes are significantly enriched in functions related to RNA metabolism and stability regulation. This may be one of the factors contributing to the stable presence of the majority of transcripts. Additionally, we have only conducted a comparative analysis between KAS-seq data and the transcripts already present in the cytoplasm. Subsequent investigations will require more optimized techniques to assess the correlation and differences between newly transcribed transcripts and the data obtained from KAS-seq. This will provide a more direct reflection of the transcriptional differences captured by KAS-seq in oocytes with different chromatin configurations.

In summary, our study employed KAS-seq to detect ssDNA signals during the oocyte meiotic maturation, capturing the transcriptional features of oocytes at various stages. The obtained results provide direct evidence for the transcriptional quiescence of MII oocytes by indicating the absence of ssDNA as a prerequisite for transcription. Furthermore, our findings also reveal that certain genes crucial for meiotic division are continuously transcribed in SN oocytes, offering a novel perspective and avenue for a deeper understanding of oocyte meiotic maturation.

### Supplementary Information

**Additional file 1: Figure S1. **KAS-seq data validation. A-D. Fingerprint plot of KAS-seq libraries and the corresponding inputs in NSN (A), SN (B), GVBD (C) and MII (D) oocytes, respectively.

**Additional file 2: Figure S2. **Validation of KAS-seq replicates. Scatter plots showing the Pearson’s correlation between KAS-seq replicates in SN, NSN, GVBD and MII oocytes.

**Additional file 3: Figure S3. **Analysis of KAS-seq peaks in GVBD oocyte and MII oocyte. A. Representation of the genomic locations of the six peaks in MII-stage oocytes. B. The distribution of KAS-seq peaks identified by KAS-seq across the genome was analyzed in GVBD oocytes. C. A snapshot from UCSC browser views showing KAS-seq peaks in GVBD oocytes.

**Additional file 4: Figure S4. **Analysis of transcriptional activity in SN and NSN oocytes. A. Methods of distinguishing SN and NSN and integrative genomics analysis. B. Analysis of nuclear configuration distinctions (SN and NSN) in GV oocytes of 3-week and 6-week-Old mice. M: Intermediate (M) type; HD: hard to discern. C. Analysis of EU staining for transcriptional activity in SN and NSN oocytes. Scale bar, 10μm. D. Bar chart showing the results of EU staining. *** *p*<0.0001. E. Dot plot showing the enriched gene ontology terms for genes expressed in SN and NSN oocytes.

**Additional file 5: Figure S5. **Analysis of the correlation between transcriptome and KAS-seq data. A. Heatmap shows the Pearson’s correlation coefficients of transcriptome in SN and NSN oocytes. B. Heatmap shows the Pearson’s correlation coefficients of KAS-seq data in SN and NSN oocytes. C. Heatmaps showing the dynamics of KAS-seq signals between SN and NSN oocytes. Genes are clustered into four groups by *k*-means algorithms.

**Additional file 6. **

**Additional file 7. **
