# Supplementary figures and images for: KAS-seq profiling captures transcription dynamics during oocyte maturation
Source: J Ovarian Res. 2024 Jan 24;17:23. doi: 10.1186/s13048-023-01342-8 (PMC10807090; doi:10.1186/s13048-023-01342-8)

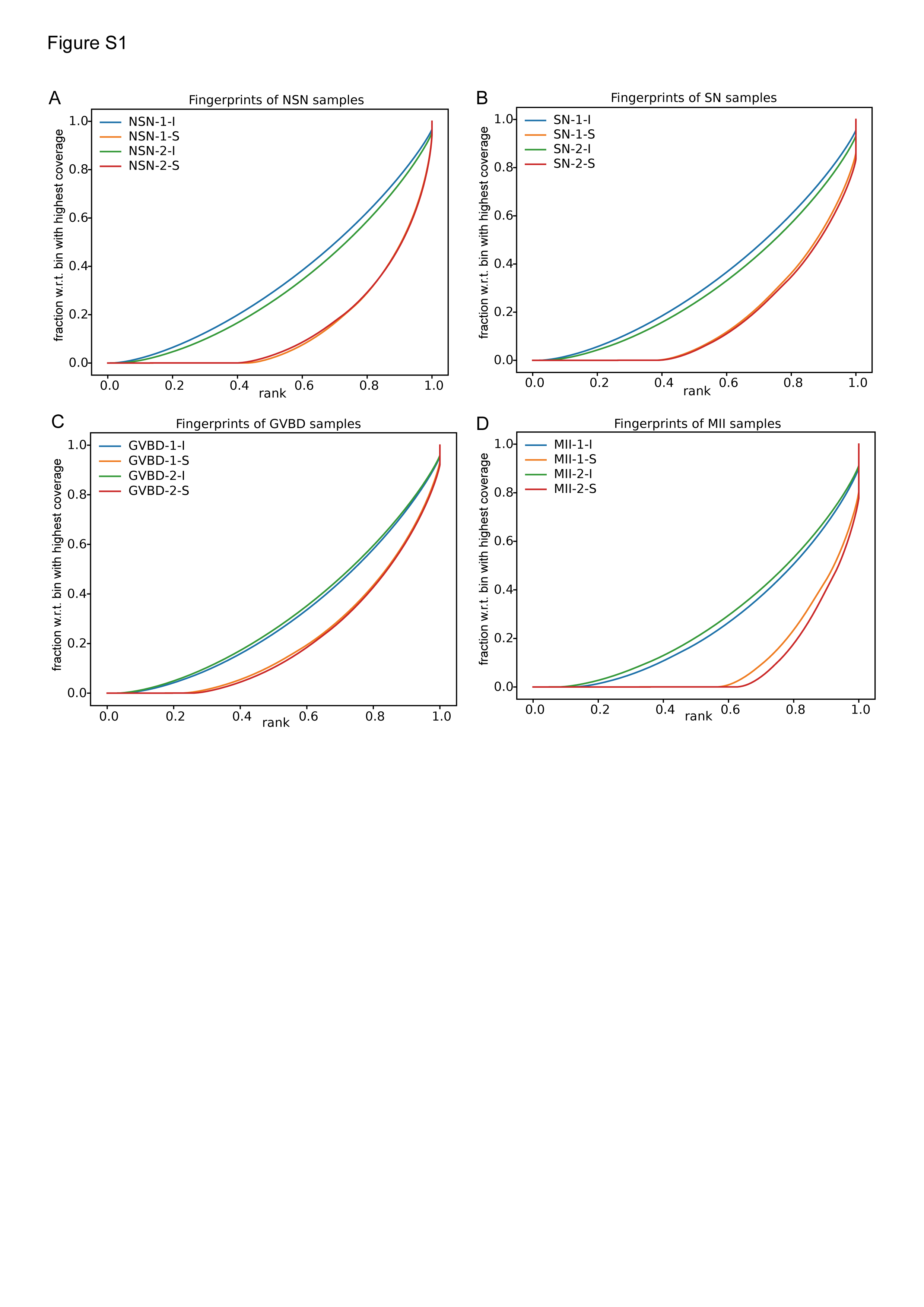

Supplement: Supplementary file 1 — Additional file 1: Figure S1. KAS-seq data validation. A-D. Fingerprint plot of KAS-seq libraries and the corresponding inputs in NSN (A), SN (B), GVBD (C) and MII (D) oocytes, respectively. [file 13048_2023_1342_MOESM1_ESM.png]

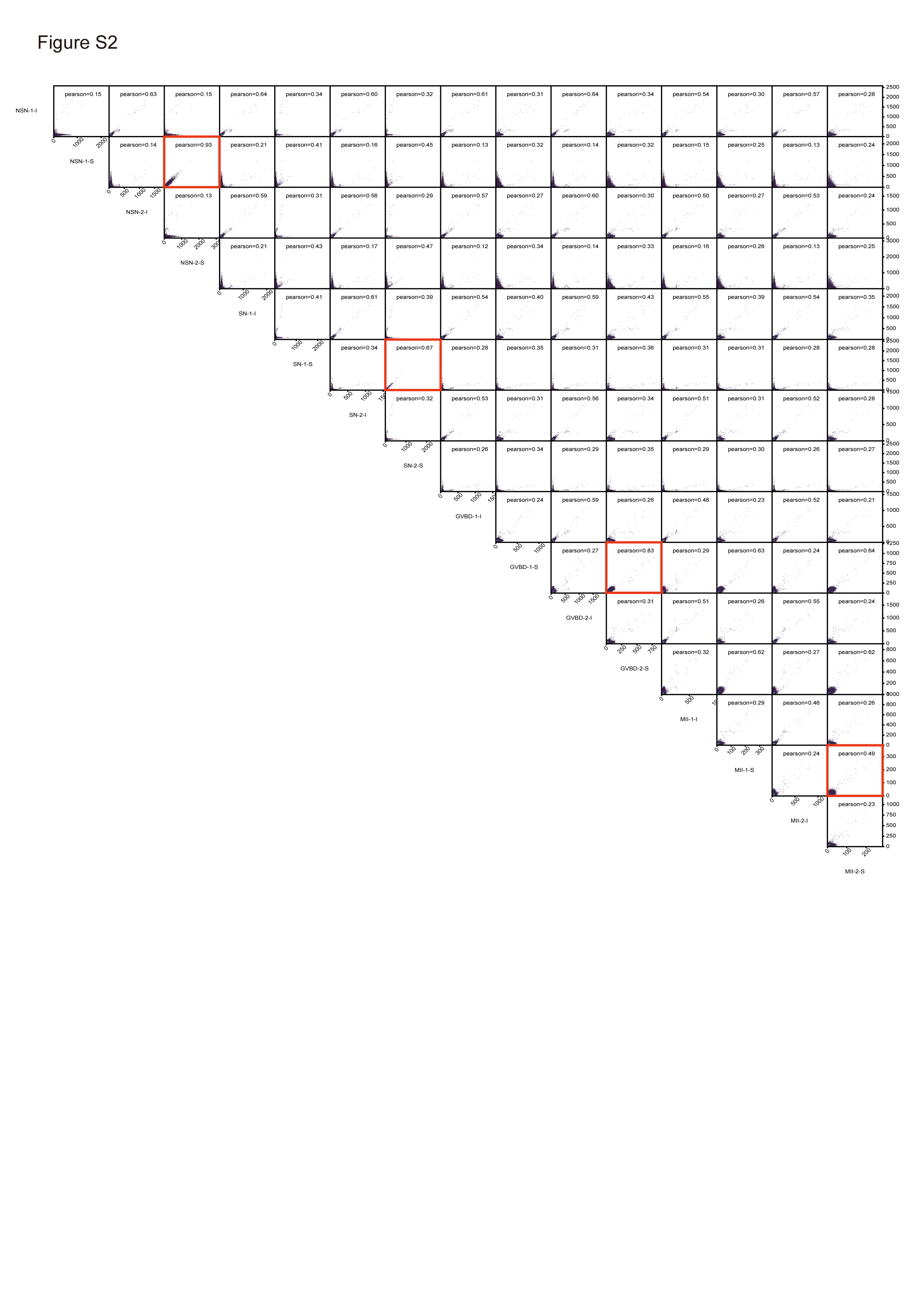

Supplement: Supplementary file 2 — Additional file 2: Figure S2. Validation of KAS-seq replicates. Scatter plots showing the Pearson’s correlation between KAS-seq replicates in SN, NSN, GVBD and MII oocytes. [file 13048_2023_1342_MOESM2_ESM.png]

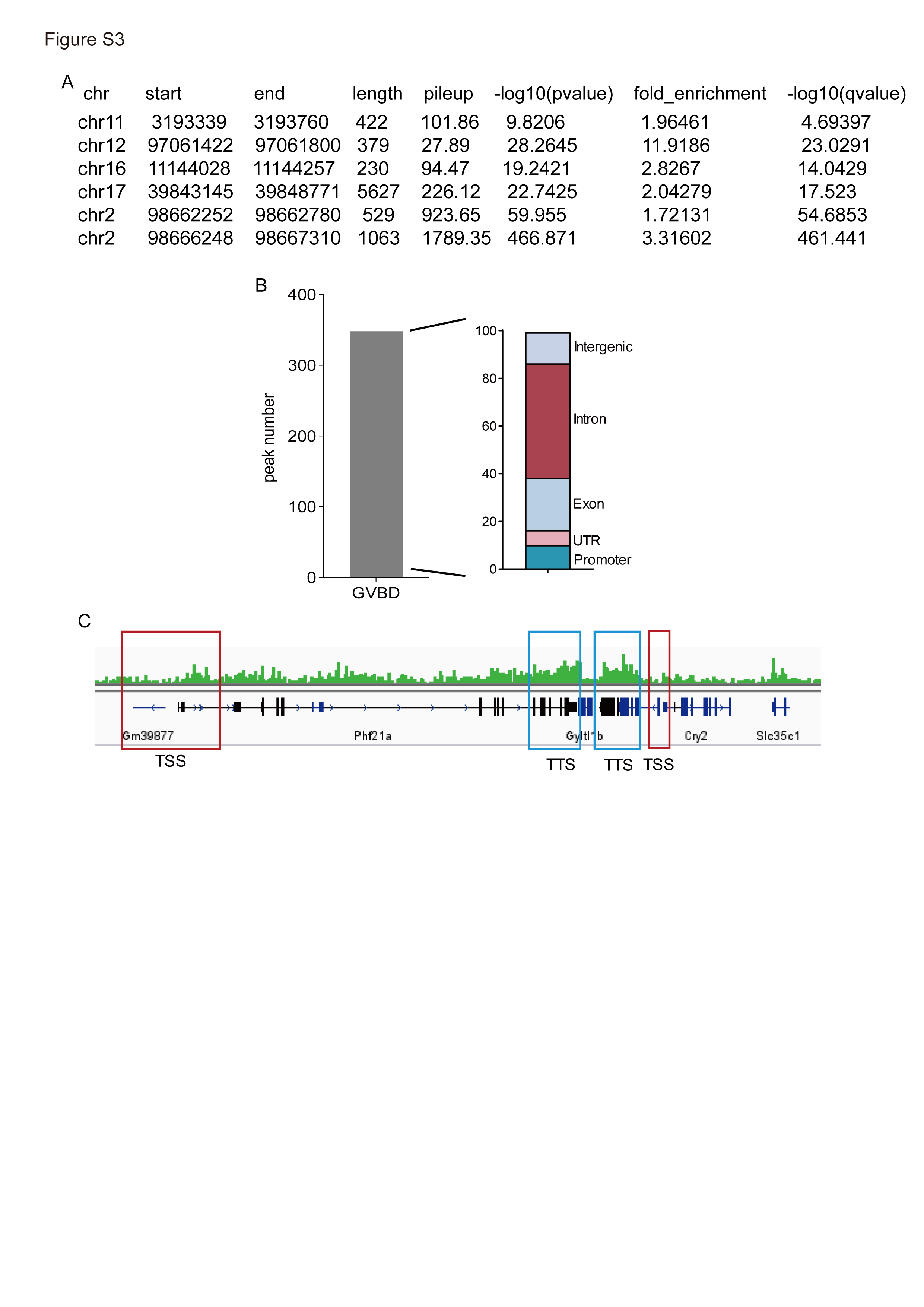

Supplement: Supplementary file 3 — Additional file 3: Figure S3. Analysis of KAS-seq peaks in GVBD oocyte and MII oocyte. A. Representation of the genomic locations of the six peaks in MII-stage oocytes. B. The distribution of KAS-seq peaks identified by KAS-seq across the genome was analyzed in GVBD oocytes. C. A snapshot from UCSC browser views showing KAS-seq peaks in GVBD oocytes. [file 13048_2023_1342_MOESM3_ESM.png]

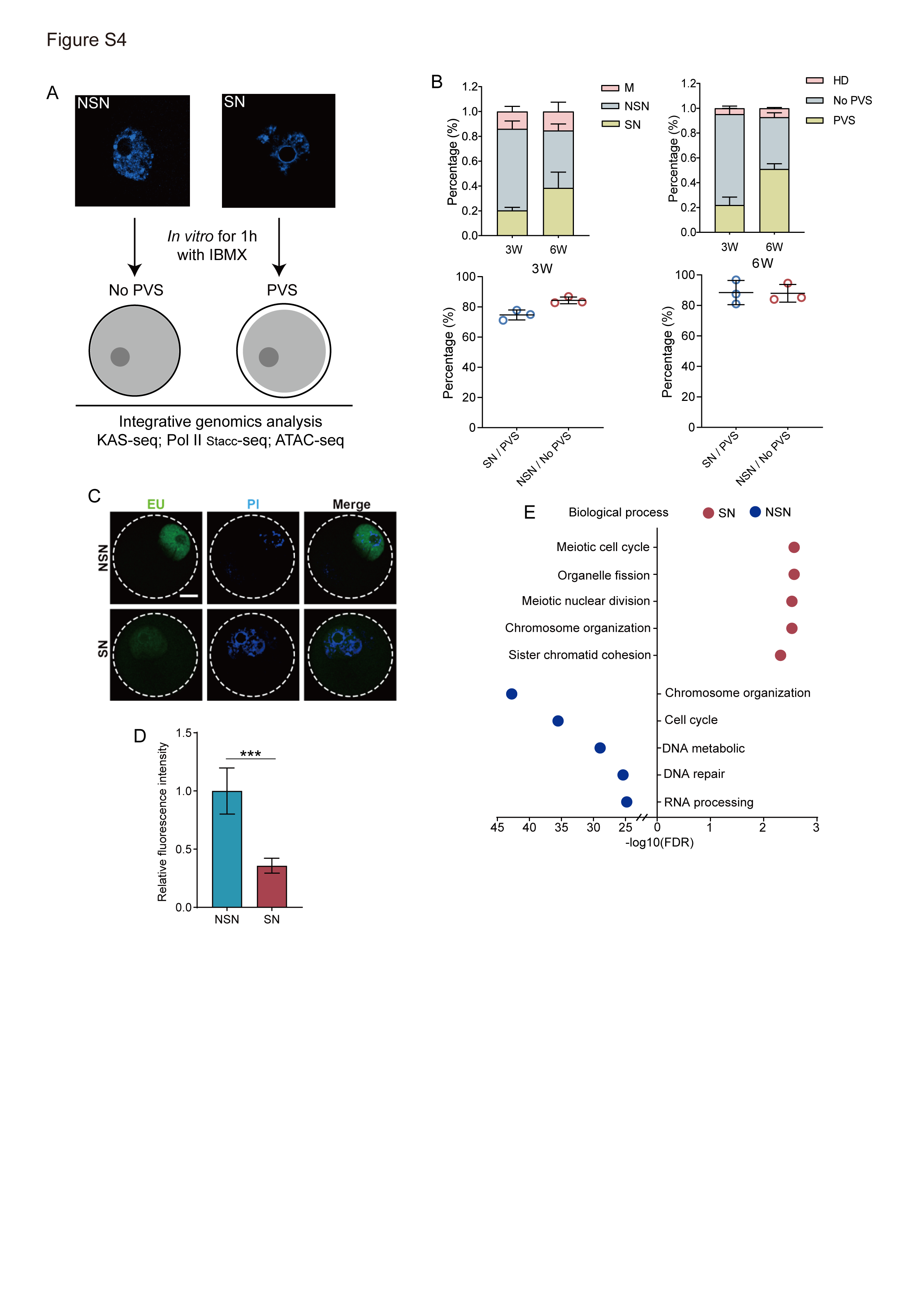

Supplement: Supplementary file 4 — Additional file 4: Figure S4. Analysis of transcriptional activity in SN and NSN oocytes. A. Methods of distinguishing SN and NSN and integrative genomics analysis. B. Analysis of nuclear configuration distinctions (SN and NSN) in GV oocytes of 3-week and 6-week-Old mice. M: Intermediate (M) type; HD: hard to discern. C. Analysis of EU staining for transcriptional activity in SN and NSN oocytes. Scale bar, 10μm. D. Bar chart showing the results of EU staining. *** p<0.0001. E. Dot plot showing the enriched gene ontology terms for genes expressed in SN and NSN oocytes. [file 13048_2023_1342_MOESM4_ESM.png]

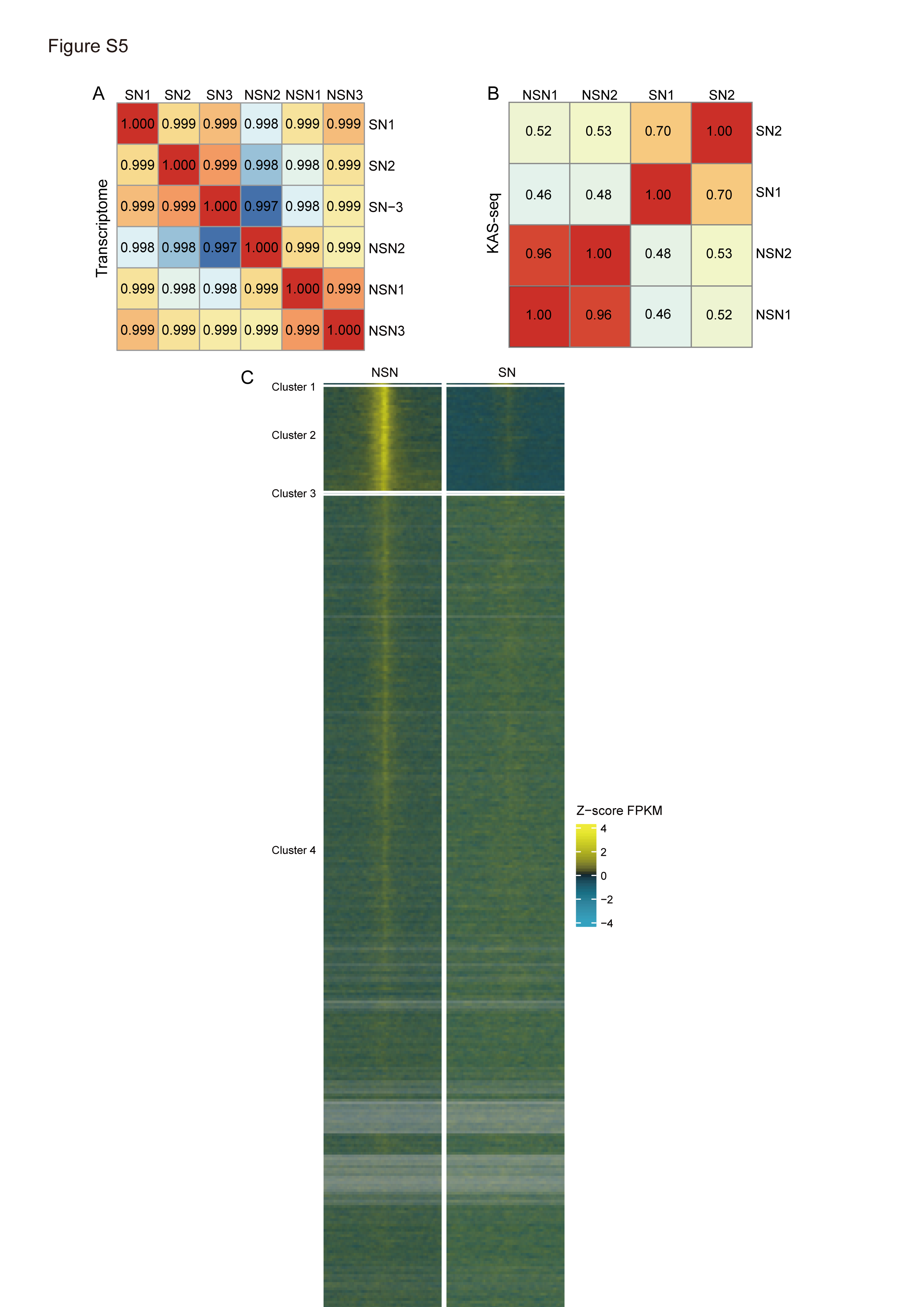

Supplement: Supplementary file 5 — Additional file 5: Figure S5. Analysis of the correlation between transcriptome and KAS-seq data. A. Heatmap shows the Pearson’s correlation coefficients of transcriptome in SN and NSN oocytes. B. Heatmap shows the Pearson’s correlation coefficients of KAS-seq data in SN and NSN oocytes. C. Heatmaps showing the dynamics of KAS-seq signals between SN and NSN oocytes. Genes are clustered into four groups by k-means algorithms. [file 13048_2023_1342_MOESM5_ESM.png]
